# Dosing practice of low molecular weight heparins and its efficacy and safety in cardiovascular inpatients: a retrospective study in a Chinese teaching hospital

**DOI:** 10.1186/1471-2261-12-118

**Published:** 2012-12-05

**Authors:** Huimin Xu, Hongwen Cai, Zhongshu Qian, Geng Xu, Xiaofeng Yan, Haibin Dai

**Affiliations:** 1 Department of Pharmacy, Second Affiliated Hospital, Zhejiang University School of Medicine, Hangzhou, 310009, China; 2 Department of Cardiovascular Medicine, First Affiliated Hospital, Zhejiang Chinese Medical University, Hangzhou, 310006, China; 3 Department of Cardiovascular Medicine, Second Affiliated Hospital, Zhejiang University School of Medicine, Hangzhou, 310009, China

**Keywords:** Low molecular weight heparin, Dosing practice, Cardiovascular inpatients, Efficacy, Safety

## Abstract

**Background:**

Low-molecular-weight heparins (LMWHs) are safe and effective anticoagulant options for cardiovascular patients when applied as body weight-adjusted doses. However, there are some barriers that make it difficult to implement weight-adjusted doses in clinical practice. Therefore, it is vital to learn the dosing practices of LMWH and its efficacy and safety in clinical practice.

**Methods:**

A retrospective study was conducted in cardiovascular inpatients who had received at least one dose of LMWH during a 6-month period. Appropriateness of LMWH dosing was determined and major clinical outcomes (major adverse vascular events and major bleeding) during hospitalization were evaluated.

**Results:**

A total of 376 admissions representing 364 patients received LMWH treatment. Of these, 17.0% (64/376) of admissions did not have body weight records. Of the 312 admissions included for the outcome study, only 34 cases (10.9%) received the recommended doses of LMWH, while 51 cases (16.3%) received mild underdoses, 223 cases (71.5%) received major underdoses and 4 (1.3%) received excess doses. There were 10 major adverse vascular events, which occurred more often in patients receiving excess doses of LMWH than in patients receiving recommended, mild or major underdoses (50%, 2.9%, 2.0% and 2.7%, respectively, *P* < 0.001). After multivariable analysis, severe renal insufficiency was an independent risk factor for major adverse vascular events [odds ratio (OR), 31.93; 95% confidence interval (CI), 5.99-170.30; *P* < 0.001]. No major bleeding was recorded.

**Conclusions:**

Underdose of LMWH is commonly used in cardiovascular inpatients, which was suboptimal according to guidelines. Using LMWH at a fixed, low dose for treatment purposes in patients without severe renal insufficiency was not associated with a higher risk of adverse vascular events in the current study, though larger studies with extended follow-ups are required to fully assess the long-term consequences of LMWH underdosing.

## Background

Cardiovascular diseases are the leading cause of morbidity and mortality in the world. Thrombosis is the final biological evolution of the atherosclerotic process, which promotes the development and progression of cardiovascular diseases [1]. In recent years several clinical trials have established, that low molecular weight heparins (LMWHs) are safe and effective anticoagulant options for patients with venous thromboembolism (VTE), acute coronary syndrome (ACS), pulmonary embolism, unstable angina and non-ST-segment elevation myocardial infarction [2-5]. This is partly due to the fact that LMWHs have superior pharmacokinetic properties as compared to unfractionated heparin (UFH) and without the need for routine coagulation tests [6]. Therefore, LMWHs have replaced UFH in most situations [7]. However, LMWHs have a longer half-life than UFH, with no potential for full reversal. Thus, if an excess dose of LMWH is given, it may result in equal or more devastating outcome than UFH. LMWHs are typically administered for embolism therapy, based on body weight, creatinine clearance and age (≥75 years) [6]. Appropriate dosing of LMWH is vital for its efficacy and safety. Previous data have shown a relationship between LMWH dose, the intensity of anticoagulation and incidence of major hemorrhage, including intracranial bleeding [8].

However, there are some barriers existing in “real world” clinical practice that make adherence to weight-adjusted doses, according to the dose-finding trials difficult [9-11]. First, accurate weight assessment is a challenge for seriously ill patients. Second, due to the high concentration of pre-filled doses of LMWH, precise measurement of a weight-based dose is difficult; this could lead to an increase in medical errors and drug waste [12]. Third, patients in real-world cardiovascular units tend to be older, have more comorbidity and are taking more prescribed drugs compared with those in clinical trials [13]. Therefore, in China, some cardiologists tend to use a lower, fixed dose of LMWH relative to the dose suggested by Chinese guidelines and recommended from the clinical trials, to reduce the perceived risk of hemorrhage and to simplify the dosing regimen. However, to our knowledge, the efficacy and safety of this “real word” clinical practice has not yet been studied.

In this retrospective study from a Chinese teaching hospital, we examined the dosing practice of LMWHs, and then determined the efficacy and safety of this practice in cardiovascular inpatients discharged from Jan 1 to Jun 30, 2010.

## Methods

### Study subjects

**Figure 1:**
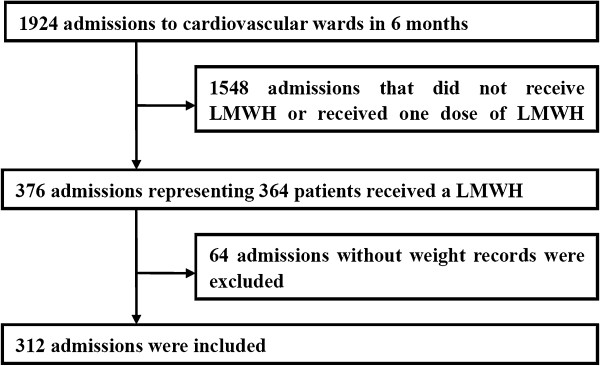
**Study design and flow chart.** Abbreviations: LMWH, low molecular weight heparin.

This study was conducted in a general, university-affiliated, teaching hospital with 2,200 licensed inpatient beds, and approximately 77,000 admissions per year. Using an electronic medical records database, the patients that were > 18 years of age and who were discharged from cardiovascular wards between Jan 1 to Jun 30, 2010 were selected. Multiple admissions of a single patient were counted as separate events. An admission was excluded if the patient did not receive any LMWH agents, received a single dose of LMWH, or had no weight record (Figure 1). The study protocol was approved by the medical ethics committee of the Second Affiliated Hospital, Zhejiang University School of Medicine, China (No. 2012–37).

Three types of LMWHs were used in this hospital: Clexane® (Enoxaparin Sodium Injection, Aventis Intercontinental, 0.4ml: 4000AxaIU), Fraxiparine® (Nadroparin Calcium Injection, GlaxoSmithKline Inc, 0.4ml: 4100AxaIU), and Fragmin® (Dalteparin Sodium Injection, Pfizer Inc, 0.2ml: 5000AxaIU) (Table 1). All LMWHs were administered by nurses during the hospitalization.

**Table 1:** **Three types of low**-**molecular**-**weight heparins used**

**Drug**	**Recommended usage**	**Individual adjustment**
Enoxaparin	100 IU/kg, every 12 h	1. 75% of the recommended dose, every 12 hours (age ≥75 years); 2. 50% of the recommended dose, every 24 hours (creatinine clearance <30 ml/min).
Nadroparin	86 IU/kg, every 12 h	
Dalteparin	120 IU/kg, every 12 h	

### Data collection and definition

A chart review was conducted for each patient included in the study. Data included basic patient demographics (gender, age, weight, and height), clinical parameters (blood creatinine, diagnosis and treatment), dosage of LMWH, and clinical outcomes (major adverse vascular events and major bleeding) during the hospitalization. The body-mass index (BMI) was calculated by dividing the individual’s weight (in kg) by the square of his or her height (in meters). Hypertension was determined by blood pressure > 140/90 mmHg or current use of antihypertensive medication. Severe renal insufficiency (RI) was determined by creatinine clearance <30 ml/min. Creatinine clearance was estimated by the Cockcroft-Gault equation [140 - age (years)] × weight (kg) × (0.85 if female) / [72 × serum creatinine (mg/dl)]. Medication orders were evaluated with the initial dose prescribed. Dosing errors were determined strictly on the initial mg/kg/dose. Interval frequency for LMWH was also collected, which was adjusted by creatinine clearance. The recommended LMWH dosage was defined in accordance with product package inserts (Table 1). Because clinical evidence of dosing strategies for nadroparin and dalteparin in patients with RI and elderly patients (≥75 years) are limited, we used the dosing strategies recommended from the data on the use of enoxaparin [14]. The following dosing categories were defined: underdose, recommended dose, and excess dose. An underdose or excess dose was defined as ≤90% or ≥110% of the recommended dose (in mg/kg/day), respectively. The underdose was further divided into mild underdose (≤90% but >80% of the recommended dose, in mg/kg/day) and major underdose (≤80% of the recommended dose, in mg/kg/day).

The efficacy and safety of clinical outcomes were evaluated by major adverse vascular events and major bleeding, respectively. Major adverse vascular events were defined as any of the following complications during in-hospital: death, myocardial re-infarction, recurrent angina, revascularization procedures, ischemic stroke, peripheral or visceral embolism, recurrent deep vein thrombosis or pulmonary embolism. Major bleeding was defined as any intracranial hemorrhage, transfusion of at least 2 units of packed red blood cells, or absolute drop in hematocrit of at least 12%; these parameters were similar to definitions used in other trials and registries [15].

### Statistical analyses

Continuous variables are reported as mean ± SD, and categorical variables are reported as numbers (percentage). Significance was determined using *χ*^2^ tests for categorical variables and Student’s t-tests or one way analysis of variance (ANOVA) for continuous data variables.

Independent predictors of major adverse vascular events were identified by use of univariable and multivariable logistic regression analysis. Important baseline characteristics such as age, gender, weight, creatinine clearance, hypertension, diabetes mellitus and dosage subgroup of LMWH were entered into the model. The odds ratio (OR) and corresponding 95% confidence interval (CI) were reported for each variable in the model. Variables were retained in the final multivariable model if their level of significance was ≤0.05.

## Results

### Patient characteristics

A total of 19.5% (376/1,924) of admissions representing 364 patients in the cardiovascular units received LMWH during this period. Of these, 17.0% (64/376) were lacking body weight records. Thus 312 admissions were analyzed for outcome analysis. Of these, 4.8% (15/312) were treated with dalteparin, 24.4% (76/312) were treated with enoxaparin, and 70.8% (221/312) were treated with nadroparin. As shown in Table 2, the mean age of patients was 64 ± 11 years, (75.3% were men). More than half of the patients (58.6%) received LMWHs for the treatment of unstable angina and non ST-segment elevation myocardial infarction. Of the 67 atrial fibrillation patients, 7 (10.4%) had valvular heart disease (mitral stenosis or prosthetic heart valves) and had used warfarin prior to hospitalization. Of the other 60 atrial fibrillation patients, only 2 had used warfarin prior to hospitalization although there were 30 cases identified as at “high risk” of stroke (defined as CHA2DS2-VASc score ≥ 2) [16]. Seven patients (2.2%) with stage 4 chronic kidney disease (CKD) received LMWH treatment.

**Table 2:** Patient and treatment characteristics

**Patient characteristics**	**Data**
Age, y	63.8 ± 11.4
≥75 years, n (%)	65 (20.8)
Female, n (%)	77 (24.7)
Body mass index	23.8 ± 3.2
Weight, kg	66.8 ± 10.9
Diagnosis, n (%)	
ST-segment elevation myocardial infarction	53 (17.0)
Unstable angina/Non ST-segment elevation Myocardial infarction	183 (58.6)
Atrial fibrillation	67 (21.5)
Deep vein thrombosis	9 (2.9)
Concurrent medical conditions, n (%)	
Hypertension	194 (62.2)
Diabetes mellitus	69 (22.1)
Severe renal insufficient, n (%)	7 (2.2)
Creatinine clearance, ml/min	83.1 ± 27.8
Treatment variables, n (%)	
Cardiac catheterization	26 (8.3)
Percutaneous coronary intervention	195 (62.5)
Radiofrequency catheter ablation	44 (14.1)
Length of hospital stay, d	8.9 ± 5.8

### LMWH dosing practices

As shown in Table 3, only 34 cases (10.9%) received the recommended dose of LMWH, 51 cases (16.3%) received mild underdoses, 223 cases (71.5%) received major underdoses, and 4 cases (1.3%) received excess doses. There were no statistically significant differences in gender, diagnosis or comorbid diseases, such as hypertension and diabetes mellitus among these groups. Patients receiving excess doses of LMWH were older, weighed more, had a poorer renal function, longer administration duration and hospital stays than the patients receiving the recommended dose (*P* < 0.05). Patients receiving major underdoses of LMWH were younger and weighted more than the patients receiving the recommended doses (*P* < 0.05). None of the patients in underdoses groups had severe RI. Major adverse vascular events occurred more often in patients receiving excess doses of LMWH than in patients receiving recommended, mild or major underdoses (50%, 2.9%, 2.0% and 2.7%, respectively, *P* < 0.001). No major bleeding was recorded in the whole study population.

**Table 3:** Baseline patient characteristics for different dosages of LMWH

**Variable**	**Major underdose (n = 223)**	**Mild underdose (n = 51)**	**Recommended dose (n = 34)**	**Excess dose (n = 4)**	***P *Value**
Age, y	62.1 ± 10.7	67.0 ± 11.3	68.6 ± 12.8	77.0 ± 17.5	<0.001
≥75 years, n (%)	24 (10.8)	19 (37.3)	19 (55.9)	3 (75)	<0.001
Female, n (%)	60 (26.9)	10 (19.6)	6 (17.6)	1 (25)	0.531
Weight (kg)	70.3 ± 9.2	58.2 ± 9.1	55.8 ± 8.8	71.5 ± 9.1	<0.001
BMI	24.7 ± 2.9	21.6 ± 2.6	21.0 ± 3.1	26.2 ± 3.1	<0.001
Diagnosis, n (%)					0.167
STEMI	37 (16.6)	9 (17.6)	6 (17.6)	1 (25)	
UA/NSTEMI	126 (56.5)	34 (66.7)	21 (61.8)	2 (50)	
Atrial fibrillation	54 (24.2)	6 (11.8)	7 (20.6)	0	
DVT	6 (2.7)	2 (3.9)	0	1 (25)	
Hypertension	134 (60.1)	34 (66.7)	23 (67.6)	3 (75)	0.668
Diabetes mellitus	53 (23.8)	7 (13.7)	9 (26.5)	0	0.267
Severe RI	0	0	3 (8.8)	4 (100)	<0.001
Creatinine, mg/dl	0.8 ± 0.2	0.8 ± 0.2	1.0 ± 0.3	2.5 ± 1.0	<0.001
Creatinine clearance, ml/min	90.6 ± 26.8	69.7 ± 16.4	61.3 ±22.6	24.9 ± 5.0	<0.001
Administration duration, d	4.1 ± 2.1	3.9 ± 1.8	4.0 ± 1.6	6.5 ± 5.6	0.128
Length of hospital stay, d	8.8 ± 5.7	9.4 ± 5.8	8.2 ± 4.6	14.8 ± 16.8	0.179
Major adverse vascular events, n (%)	6 (2.7)	1 (2.0)	1 (2.9)	2 (50)	<0.001

Abbreviations: *LMWH*, Low molecular weight heparin; *BMI*, body mass index; *STEMI*, ST-segment elevation myocardial infarction; *UA/NSTEMI*, Unstable angina/Non ST-segment elevation myocardial infarction; *DVT*, Deep vein thrombosis; *RI*, renal insufficient.

### Major adverse vascular events and risk factors

There were 10 major adverse vascular events (3.2%) in this study, including four deaths, three myocardial re-infarctions, two revascularization procedures and one ischemic stroke. Major adverse vascular events occurred at 5.9 ± 9.0 days after admission. There was no difference in the major adverse vascular event rate when comparing different diagnoses (P > 0.05). Major adverse vascular events occurred in 4 patients with ST-segment elevation myocardial infarction (7.5%), 4 patients with unstable angina/non ST-segment elevation myocardial infarction (2.2%), 1 patient with atrial fibrillation (1.5%), and 1 patient with deep vein thrombosis (11.1%). Univariate analysis of the risk factors of major adverse vascular events is presented in Table 4. Multivariable analysis found that only one risk factor (severe RI) was independently associated with an increased risk for major adverse vascular events (OR, 31.93; 95% CI, 5.99-170.30; *P* < 0.001), while age≥75 years, excess doses of LMWH were not independent predictors of major adverse vascular events.

**Table 4:** **Risk factors for major adverse vascular events** (**univariable analysis**)

**Characteristics**	**N (%)**		OR (95% CI) *	***P *Value**
	**Without major adverse vascular events (n=302)**	**Major adverse vascular events (n=10)**		
Age, ≥75 y	60 (20)	5 (50)	4.03 (1.13-14.38)	0.036
Female	72 (24)	5 (50)	3.19 (0.90-11.35)	0.071
Weight, >60 kg	235 (78)	10 (100)	--	0.086
STEMI	49 (16)	4 (40)	3.44 (0.94-12.65)	0.071
Hypertension	186 (62)	8 (80)	2.50 (0.52-11.95)	0.201
Diabetes mellitus	69 (23)	0	--	0.079
Severe RI	4 (1.3)	3 (30)	31.93 (5.99-170.30)	0.001
Inappropriate dose	269 (89)	9 (90)	1.104 (0.14-8.99)	0.701
Excess dose	2 (0.7)	2 (20)	37.5 (4.68-300.75)	0.005
Underdose	267 (88)	7 (70)	0.306 (0.08-1.24)	0.109
Major underdose	217 (72)	6 (60)	0.588 (0.162-2.134)	0.310

Abbreviations: *STEMI*, ST-segment elevation myocardial infarction; *RI*, renal insufficient; *OR*, odds ratio; *CI*, confidence interval.

*Reference groups include age<75 years, male, weight ≤60 kg, no STEMI, no hypertension, no diabetes mellitus, no severe RI, recommended dose, no excess dose, no underdose, no major underdose.

## Discussion

In the present study we assessed LMWH dosing practices in 364 cardiovascular inpatients (376 admissions) and identified the efficacy and safety of LMWH treatment in 312 cases as measured by major adverse vascular events and major bleeding. There is a considerable disparity in LMWH use when comparing clinical practice to the guideline [6]. Seventeen percent of patients (64/376) without body weight records received LMWH, 10.9% (34/312) of patients received the recommended doses of LMWH and 87.8% (274/312) received underdoses of LMWH. Interestingly, we found that receiving underdoses of LMWH was not a risk factor for major adverse vascular events. The only risk factor for major adverse vascular events was severe RI.

LMWHs are prescribed based on the patient’s weight according to dose-finding studies. However, accurate weight assessment is a challenge for seriously ill patients that are due to the limitations of resources, physical space and time [17]. In our study, we found that approximately 1 in 6 patients received LMWHs without having a record of weight. This is similar to a previous study reported that approximately 1 in 10 patients received enoxaparin for treatment of an ACS without weight documentation [13]. Furthermore, it has been reported that estimation of patients’ weights by health care providers is inaccurate having a mean error of 9 to 10 kg [18]. Inappropriate dosing of LMWH can easily occur if the weight is estimated incorrectly, which can lead to medication errors in clinical practice. However, studies of the consequences of inappropriate dosing of LMWHs in real world practices are still scarce.

The major complication of anticoagulant and thrombolytic therapy is bleeding. A previous study demonstrated that patients with ACS often received excess doses of LMWH that was accompanied by an increased risk of major bleeding [15]. In order to decrease the risk of bleeding, some clinicians in China often prefer to choose empirical dose strategies when administering LMWHs instead of those reported in the results of clinical trials or suggested by Chinese guidelines. In our hospital, the most commonly adopted dosing strategies are enoxaparin 4,000AxaIU, nadroparin 4,100AxaIU or dalteparin 5,000AxaIU given twice daily when a patient’s weight is < 80 kg. For patients that weigh ≥ 80 kg, enoxaparin 6,000AxaIU, nadroparin 6,200AxaIU or dalteparin 7,500AxaIU are given twice daily. These dosing strategies reflected the reason for the high rate of LMWH underdosing in the current study. As a result, there were no major bleeding events in our study; conversely, the rate of major bleeding events has been found to be approximately 1–6.5% in clinical trials of LMWHs [9-11]. Therefore, the practice of underdosing LMWH done in our hospital appears to be safe.

Unlike excess dosing of LMWH, which is related to a risk of bleeding, foremost concern associated with underdose of LMWH is the risk of embolism. Thus, we determined the efficacy of the current dosing practice of LMWHs, as measured by the incidence of embolism. Surprisingly, in the underdose LMWH group, the incidence of major adverse vascular events was similar to that of the group receiving the recommended dose (2.6% *vs.* 2.9%). Previous data have demonstrated that patients with low anti-Xa activity increased 30-day mortality [19]. This may be due to the fact that the underdose of LMWH in our study doesn’t indicate low anti-Xa activity as LMWH has linear pharmacokinetics but high between-subject variability [20,21]. On the other hand, clinical outcomes are also influenced by patients’ characteristics and not only the dosage of LMWH. In the above mentioned study, patients with sub-therapeutic anti-Xa levels were significantly older, and had more impaired renal function and inferior heart function compared with others [19]. However, in our study, patients that received underdoses of LMWH had better baseline characteristics (younger and better renal function) than the recommended and excess dose groups; this may be the primary reason that the underdosing of LMWH was not found to be a risk factor for embolism in our study.

Since there were no severe RI patients receiving underdoses of LMWH, a fixed and weight-independent dosage may be not suitable for severe RI patients. LMWHs are not recommended for use in patients with severe RI due to the risk of accumulation may lead to major bleeding [11,22,23]. According to the results of the ExTRACT-TIMI 25 trial [11], a dose of 1 mg/kg of enoxaparin every 24 h was recommended for patients with an estimated creatinine clearance <30 ml/min. However, in our study, major adverse vascular events occurred more often in severe RI patients than in patients with creatine clearance ≥30 ml/min (30% *vs.* 1.3%, *P* < 0.001), although 57.1% (4/7) of severe RI patients receiving excess doses of LMWH. After multivariable analysis, we found that severe RI is the only predictor of major adverse vascular events. In elderly patients (≥75 years), STEMI patients, excess doses of LMWH were not independent predictors of major adverse vascular events. Results from the current study are consistent with those from previous studies in which patients with RI have a higher risk for thromboembolic complications [24-26], and support the recommendation for dose adjustments based on anti-Xa activity and not calculated based on a simple dose scheme for LMWH used in RI patients [27]. Our results indicate that using LMWH at a fixed, lower dose in patients without severe RI may be safe and effective.

Nevertheless, there are some limitations to this study. First, our findings are based on the results of a one-center, retrospective study, complementing the prospect and lack of follow-up. Second, unlike randomized trials that have more restrictive inclusion criteria, it must be noted that the patient characteristics in this study were not as well-controlled. Third, due to a lack of anti-Xa assay in our hospital, we could not measure anti-Xa activity in RI patients. Finally, small sample size is a potential limiting factor in this study as well.

## Conclusions

In summary, the current study demonstrated that underdose of LMWH is commonly used in cardiovascular inpatients. Using LMWH in a fixed, lower dose for treatment purposes in patients without severe RI was not associated with a high risk of adverse vascular events in our study. Larger studies with extended follow-ups are required to fully assess the long-term consequences of LMWH underdosing.

## Competing interests

The authors declare that there are no conflicts of interest regarding this study to be disclosed.

## Authors’ contributions

HX, HC and ZQ carried out the medical records database search, statistical analysis, and drafted the manuscript. GX, XY, and HD participated in the design and coordination of the study and helped to draft the manuscript. All authors read and approved the final manuscript.

## Pre-publication history

The pre-publication history for this paper can be accessed here:

http://www.biomedcentral.com/1471-2261/12/118/prepub

## References

[B1] Michaels AD Spinler SA Leeper B Ohman EM Alexander KP Newby LK Ay H Gibler WB Medication errors in acute cardiovascular and stroke patients: a scientific statement from the American Heart Association Circulation 2010 121 14 1664 1682 10.1161/CIR.0b013e3181d4b43e 20308619

[B2] Hirsh J Warkentin TE Shaughnessy SG Anand SS Halperin JL Raschke R Granger C Ohman EM Dalen JE Heparin and low-molecular-weight heparin: mechanisms of action, pharmacokinetics, dosing, monitoring, efficacy, and safety Chest 2001 119 1 Suppl 64S 94S 11157643 10.1378/chest.119.1_suppl.64s

[B3] Erkens PM Prins MH Fixed dose subcutaneous low molecular weight heparins versus adjusted dose unfractionated heparin for venous thromboembolism Cochrane Database Syst Rev 2010 9 CD001100 20824828 10.1002/14651858.CD001100.pub3

[B4] Magee KD Sevcik W Moher D Rowe BH Low molecular weight heparins versus unfractionated heparin for acute coronary syndromes Cochrane Database Syst Rev 2003 1 CD002132 12535430 10.1002/14651858.CD002132 PMC13459081

[B5] Font MA Krupinski J Arboix A Antithrombotic medication for cardioembolic stroke prevention Stroke Res Treat 2011 2011 607852 21822469 10.4061/2011/607852 PMC3148601

[B6] Hirsh J Raschke R Heparin and low-molecular-weight heparin: the seventh ACCP conference on antithrombotic and thrombolytic therapy Chest 2004 126 3 Suppl 188S 203S 15383472 10.1378/chest.126.3_suppl.188S

[B7] Warkentin TE Levine MN Hirsh J Horsewood P Roberts RS Gent M Kelton JG Heparin-induced thrombocytopenia in patients treated with low-molecular-weight heparin or unfractionated heparin N Engl J Med 1995 332 20 1330 1335 10.1056/NEJM199505183322003 7715641

[B8] Schulman S Beyth RJ Kearon C Levine MN Hemorrhagic complications of anticoagulant and thrombolytic treatment: american college of chest physicians evidence-based clinical practice guidelines (8th edition) Chest 2008 133 6 Suppl 257S 298S 18574268 10.1378/chest.08-0674

[B9] Cohen M Demers C Gurfinkel EP Turpie AG Fromell GJ Goodman S Langer A Califf RM Fox KA Premmereur J A comparison of low-molecular-weight heparin with unfractionated heparin for unstable coronary artery disease efficacy and safety of subcutaneous enoxaparin in Non-Q-wave coronary events study group N Engl J Med 1997 337 7 447 452 10.1056/NEJM199708143370702 9250846

[B10] The FRAX.I.S. Study Group Comparison of two treatment durations (6 days and 14 days) of a low molecular weight heparin with a 6-day treatment of unfractionated heparin in the initial management of unstable angina or non-Q wave myocardial infarction: FRAX.I.S. (FRAxiparine in Ischaemic Syndrome) Eur Heart J 1999 20 21 1553 1562 10.1053/euhj.1999.1879 10529323

[B11] Klein W Buchwald A Hillis SE Monrad S Sanz G Turpie AG van der Meer J Olaisson E Undeland S Ludwig K Comparison of low-molecular-weight heparin with unfractionated heparin acutely and with placebo for 6 weeks in the management of unstable coronary artery disease. Fragmin in unstable coronary artery disease study (FRIC) Circulation 1997 96 1 61 68 10.1161/01.CIR.96.1.61 9236418

[B12] Bauman ME Black KL Bauman ML Belletrutti M Bajzar L Massicotte MP Novel uses of insulin syringes to reduce dosing errors: a retrospective chart review of enoxaparin whole milligram dosing Thromb Res 2009 123 6 845 847 10.1016/j.thromres.2008.09.001 19038418

[B13] Macie C Forbes L Foster GA Douketis JD Dosing practices and risk factors for bleeding in patients receiving enoxaparin for the treatment of an acute coronary syndrome Chest 2004 125 5 1616 1621 10.1378/chest.125.5.1616 15136367

[B14] Fox KA Antman EM Montalescot G Agewall S SomaRaju B Verheugt FW Lopez-Sendon J Hod H Murphy SA Braunwald E The impact of renal dysfunction on outcomes in the ExTRACT-TIMI 25 trial J Am Coll Cardiol 2007 49 23 2249 2255 10.1016/j.jacc.2006.12.049 17560289

[B15] Alexander KP Chen AY Roe MT Newby LK Gibson CM Allen-LaPointe NM Pollack C Gibler WB Ohman EM Peterson ED Excess dosing of antiplatelet and antithrombin agents in the treatment of non-ST-segment elevation acute coronary syndromes JAMA 2005 294 24 3108 3116 10.1001/jama.294.24.3108 16380591

[B16] Camm AJ Kirchhof P Lip GY Schotten U Savelieva I Ernst S Van Gelder IC Al-Attar N Hindricks G Prendergast B Guidelines for the management of atrial fibrillation: the task force for the management of atrial fibrillation of the European Society of Cardiology (ESC) Eur Heart J 2010 31 19 2369 2429 20802247 10.1093/eurheartj/ehq278

[B17] Lin BW Yoshida D Quinn J Strehlow M A better way to estimate adult patients' weights Am J Emerg Med 2009 27 9 1060 1064 10.1016/j.ajem.2008.08.018 19931751

[B18] Anglemyer BL Hernandez C Brice JH Zou B The accuracy of visual estimation of body weight in the ED Am J Emerg Med 2004 22 7 526 529 10.1016/j.ajem.2004.09.002 15666254

[B19] Montalescot G Collet JP Tanguy ML Ankri A Payot L Dumaine R Choussat R Beygui F Gallois V Thomas D Anti-Xa activity relates to survival and efficacy in unselected acute coronary syndrome patients treated with enoxaparin Circulation 2004 110 4 392 398 10.1161/01.CIR.0000136830.65073.C7 15249498

[B20] Al-Sallami HS Barras MA Green B Duffull SB Routine plasma anti-Xa monitoring is required for low-molecular-weight heparins Clin Pharmacokinet 2010 49 9 567 571 10.2165/11532960-000000000-00000 20690780

[B21] Singer JP Huang MY Hui C Blanc PD Boettger RF Golden J Watkins K Hoopes C Leard LE Supratherapeutic anticoagulation from low-molecular-weight heparin in lung transplant recipients J Heart Lung Transplant 2010 29 9 1009 1013 10.1016/j.healun.2010.04.018 20627627 PMC3045833

[B22] Bazinet A Almanric K Brunet C Turcotte I Martineau J Caron S Blais N Lalonde L Dosage of enoxaparin among obese and renal impairment patients Thromb Res 2005 116 1 41 50 10.1016/j.thromres.2004.10.004 15850607

[B23] Schmid P Fischer AG Wuillemin WA Low-molecular-weight heparin in patients with renal insufficiency Swiss Med Wkly 2009 139 31–32 438 452 19685350 10.4414/smw.2009.11284

[B24] Frydman A Low-molecular-weight heparins: an overview of their pharmacodynamics, pharmacokinetics and metabolism in humans Haemostasis 1996 26 Suppl 2 24 38 8707165 10.1159/000217270

[B25] Jacobs DR Jr Kroenke C Crow R Deshpande M Gu DF Gatewood L Blackburn H PREDICT: A simple risk score for clinical severity and long-term prognosis after hospitalization for acute myocardial infarction or unstable angina: the Minnesota heart survey Circulation 1999 100 6 599 607 10.1161/01.CIR.100.6.599 10441096

[B26] Collet JP Montalescot G Fine E Golmard JL Dalby M Choussat R Ankri A Dumaine R Lesty C Vignolles N Enoxaparin in unstable angina patients who would have been excluded from randomized pivotal trials J Am Coll Cardiol 2003 41 1 8 14 10.1016/S0735-1097(02)02664-5 12570937

[B27] Schmid P Brodmann D Odermatt Y Fischer AG Wuillemin WA Study of bioaccumulation of dalteparin at a therapeutic dose in patients with renal insufficiency J Thromb Haemost 2009 7 10 1629 1632 10.1111/j.1538-7836.2009.03556.x 19624460

